# Journal of Oral Microbiology extends its editorial competence in immunology promoting its international impact

**DOI:** 10.1080/20002297.2018.1485819

**Published:** 2018-06-21

I have the pleasure to announce that Greg Seymour, Emeritus Professor, The University of Queensland, Australia, and John Taylor, Senior Lecturer in Molecular Immunology, School of Dental Sciences, Newcastle University, UK are new Associate Editors in the Journal of Oral Microbiology. Their major field will be immunology.

I am also happy to tell that Professors Ann Progulske-Fox, College of Dentistry, University of Florida, and Bruce J Paster, Forsyth Institute/Harvard School of Dental Medicine will still be involved as Associate Editors in the journal in the field of microbiology.

Professor Philip Marsh has retired from his post at Public Health England and has therefore decided to step down from his role as Associate Editor in Journal of Oral Microbiology. I will miss him deeply as co-worker and thank him for his generous work for the journal over the years.

Oslo, June 1, 2018

Ingar Olsen

Editor-in-Chief

